# PEDOT:PSS versus Polyaniline: A Comparative Study of Conducting Polymers for Organic Electrochemical Transistors

**DOI:** 10.3390/polym15244657

**Published:** 2023-12-10

**Authors:** Ryotaro Kawamura, Tsuyoshi Michinobu

**Affiliations:** Department of Materials Science and Engineering, Tokyo Institute of Technology, 2-12-1 Ookayama, Meguro-ku, Tokyo 152-8552, Japan; kawamura.r.ah@m.titech.ac.jp

**Keywords:** organic electrochemical transistor, PEDOT:PSS, PANI, film morphology

## Abstract

Organic electrochemical transistors (OECTs) based on conducting polymers have attracted significant attention in the field of biosensors. PEDOT:PSS and polyaniline (PANI) are representative conducting polymers used for OECTs. While there are many studies on PEDOT:PSS, there are not so many reports on PANI-based OECTs, and a detailed study to compare these two polymers has been desired. In this study, we investigated the fabrication conditions to produce the best performance in the OECTs using the above-mentioned two types of conducting polymers. The two main parameters were film thickness and film surface roughness. For PEDOT:PSS, the optimal conditions for fabricating thin films were a spin-coating rate of 3000 rpm and a DI water immersion time of 18 h. For PANI, the optimal conditions were a spin-coating rate of 3000 rpm and DI water immersion time of 5 s, and adding dodecylbenzenesulfonic acid (DBSA) was found to provide better OECT performances. The OECT performances based on PEDOT:PSS were superior to those based on PANI in terms of conductivity and transconductance, but PANI showed excellence in terms of film thickness and surface smoothness, leading to the good reproducibility of OECT performances.

## 1. Introduction

Conducting polymers are expected to find applications in various industrial fields because of their light weight, low cost, and flexibility [1,2,3,4,5,6,7,8,9]. They become conductive by either chemical or electrochemical doping, which injects charge carriers into polymers [10]. Doping allows the conductivity of polymers to be freely controlled, as well as other physical properties. Poly(3,4-ethylenedioxythiophene) polystyrene sulfonate (PEDOT:PSS) is one of the most studied conducting polymers, and its applications in sensing and other technologies, such as pH sensors [11], glucose sensors [12], and enzyme sensors [13], are in practical use [14]. This is partly because conducting polymers are a good platform for the functionalization of many biological molecules and the enhancement of biosensor performances. In addition, functional groups of organic polymers have many weak intermolecular interactions. Controlling such intermolecular interactions enables the enhancement of the stability and sensitivity of biosensors. Moreover, conducting polymers can be combined with other materials, such as metals and metal oxides, to form composites. Synergistic effects of sensor properties by composite materials are highly expected.

PEDOT:PSS is usually dispersed as colloids in an aqueous solution, and the thin films can be prepared by solution casting. It is known that the conductivity of PEDOT:PSS increases by nearly two orders of magnitude by adding specific solvents, such as ethylene glycol, dimethyl sulfoxide, and sorbitol [15]. When OECT applications are considered, PEDOT:PSS films must be crosslinked because OECTs operate with aqueous electrolytes. Therefore, effective cross-linkers, such as (3-glycidyloxypropyl)trimethoxysilane (GOPS), are used to make them insoluble in aqueous solutions [16].

In addition to PEDOT:PSS, polyaniline (PANI) is a typical example of a conducting polymer. One of the main characteristics of PANI is that the raw material, aniline, is relatively inexpensive, and oxidative polymerization is easy. Furthermore, it is known that the solubility of organic solvents can be improved by using dopants such as 10-camphorsulfonic acid (CSA) and dodecylbenzenesulfonic acid (DBSA) [10]. Moreover, the raw material of aniline is biodegradable and has a low environmental impact [17]. Therefore, in recent years, PANI has been expected to be applied to the field of sensing [18]. On the other hand, it has been pointed out that the conductivity of PANI is inferior to PEDOT:PSS. Therefore, the use of PANI in the development of biosensors with good sensitivity and accuracy remains a challenge. It should be noted that the long-term stability of OECTs based on PEDOT:PSS was comprehensively investigated [19], while the study of PANI is still in its infancy and much room remains for further research.

There are many examples of OECTs based on conducting polymers [20,21,22,23,24,25,26,27,28,29]. However, further research is needed to improve their performance for use in high-performance biosensors and related technologies. In this study, OECTs based on two well-known conducting polymers, PEDOT:PSS and PANI, were fabricated, and their device performances and durability were optimized. By comparing the two types of conducting polymers, we aim to discuss more optimal conditions for OECTs using conducting polymers, especially the applicability of PANI-based OECTs, because there are few precedents [30,31].

## 2. Materials and Methods

PEDOT:PSS (0.5–1 wt% dispersion in water) was purchased from Sigma–Aldrich, Burlington, MA, USA. Polyaniline (PANI) was kindly supplied from the Idemitsu Co., Tokyo, Japan. The thin films of both polymers display visible to near-infrared absorption and no fluorescence due to the partially doped structures (Figure S1). Ethylene glycol was purchased from Kanto Chemicals Co., Tokyo, Japan. (3-Glycidyloxypropyl)trimethoxysilane (GOPS) and dodecylbenzene sulfonic acid (DBSA) were purchased from TCI. These chemicals were used to blend the PEDOT:PSS or PANI solution. For the preparation, 150 μL (3%) of ethylene glycol, 12 μL (about 0.25%) of DBSA, and 50 μL (1%) of GOPS were mixed with 5 mL of the commercial PEDOT:PSS solution. First, ethylene glycol and DBSA were added to PEDOT:PSS and stirred for 10 min with sonication. Then, 50 μL (1%) of GOPS was added and stirred for 1 min while being sonicated again. For PANI:DBSA, about 12 μL of DBSA was mixed with 5 mL of PANI and stirred with sonication.

The fabrication of OECTs was done with care to avoid contamination with impurities. First, the surface of the electrode substrate was cleaned with DI water and made hydrophilic by irradiating it with ozone plasma for 20 min. The commercial PEDOT:PSS solution was spin-coated onto the surface-modified electrode. First, the rotation speed was set to 4000 rpm, and DI water was dropped and rotated for 30 s to briefly clean the surface. After that, 75 μL of the PEDOT:PSS solution was dropped and held without rotation for 100 s. Spin coating was then performed at a constant rotation speed (1000–3000 rpm) for 40 s. After spin-coating was completed, it was annealed at 135 °C for 1 h to form a PEDOT:PSS film on the electrode. The electrode was immersed in DI water (0~18 h) to remove impurities such as low-molecular-weight PEDOT and form a smooth film surface. Coatings in unnecessary areas were removed by blowing water off the surface. The prepared samples are summarized in Table 1. In the case of PANI and PANI:DBSA, ozone plasma irradiation was not performed, and chloroform was used to clean the electrodes instead of DI water. Other than these points, the same procedure was used for spin-coating, followed by annealing at 135 °C for 30 min. Immersing in DI water was performed only for 0 s and 5 s. The prepared samples are summarized in Table 2.

In the OECT measurements, conductivity and transconductance values were calculated from the output and transfer characteristics. The electrodes used were L = 10 μm and W = 2500 μm. In the measurements of output characteristics, conductivities were calculated from the portion of the linear region with a gate voltage of 0 V. The measurement conditions were set for V_D_ from 0 V to 1.5 V and for V_G_ from 0 V to 2.0 V at 0.4 V intervals. For the measurement conditions of the transfer characteristics of the PEDOT:PSS device, V_G_ was set from 2.2 V to −1.0 V, and V_D_ was fixed at −0.6 V. The transconductance that reached its peak due to the sudden change in drain current was replaced by the value before or after the peak.

## 3. Results

### 3.1. Output Characteristics

**Figure 1:**
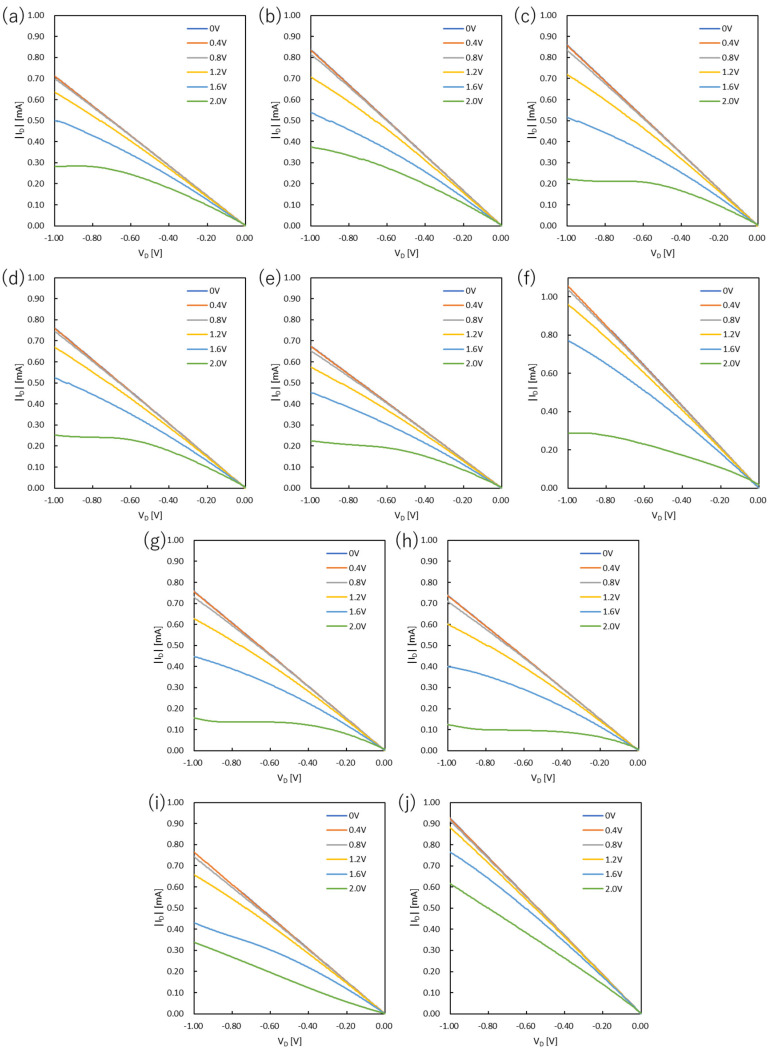
Output curves of OECTs based on PEDOT:PSS: (**a**) P1000-5 s, (**b**) P1500-5 s, (**c**) P2000-5 s, (**d**) P2500-5 s, (**e**) P3000-5 s, (**f**) P2000-0 s, (**g**) P2000-5 min, (**h**) P2000-15 min, (**i**) P2000-1 h, and (**j**) P2000-18 h.

**Figure 2:**
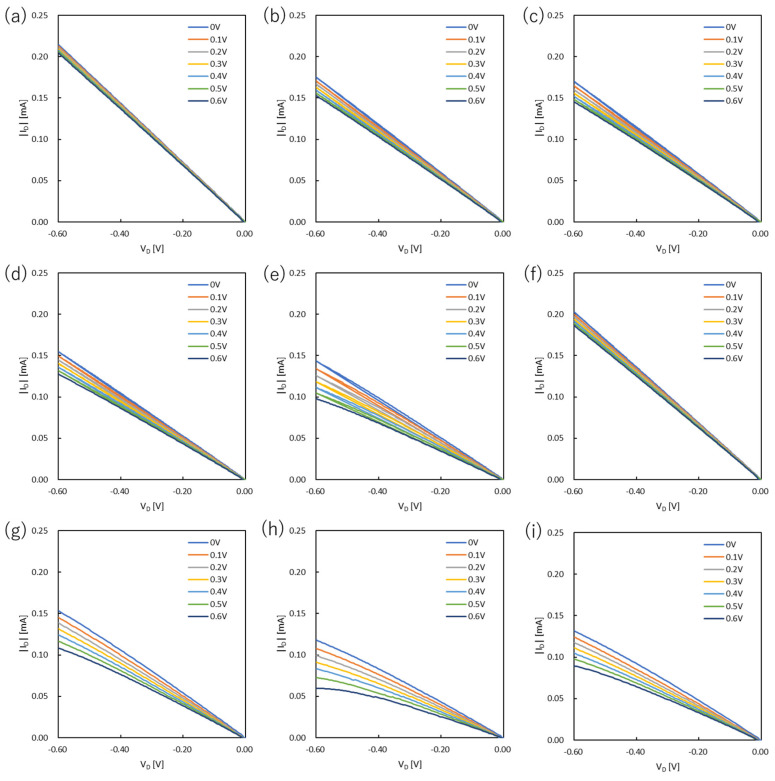
Output curves of OECTs based on PANI and PANI:DBSA: (**a**) PA1000-5 s, (**b**) PA1500-5 s, (**c**) PA2000-5 s, (**d**) PA2500-5 s, (**e**) PA3000-5 s, (**f**) PA1500-0 s, (**g**) PAD1500-5 s, (**h**) PAD3000-5 s, and (**i**) PAD1500-0 s. PA and PAD represent PANI and PANI:DBSA, respectively.

**Figure 3:**
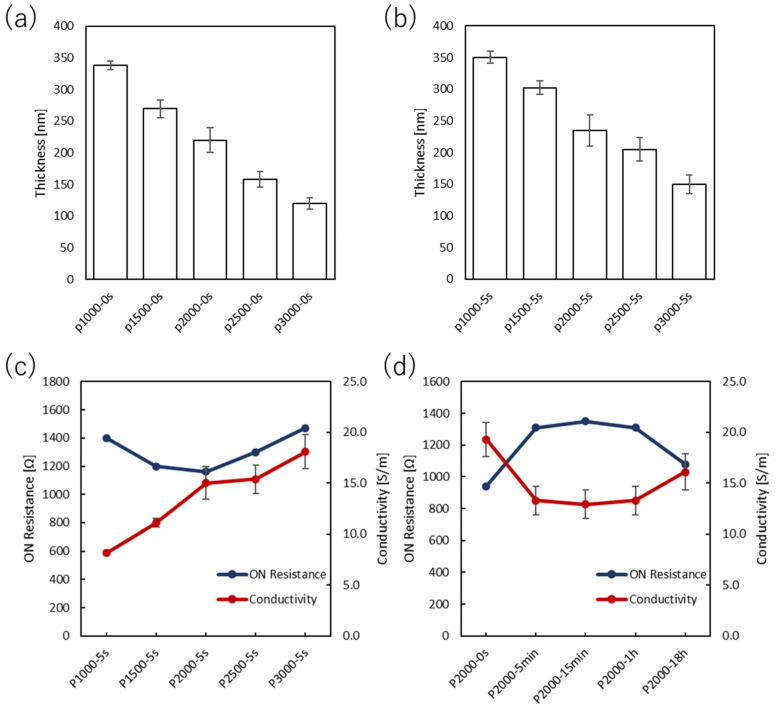
Film thicknesses of PEDOT:PSS OECTs at various spin-coating rates with an immersion time of (**a**) 0 s and (**b**) 5 s. The on-resistance and conductivity values at (**c**) various spin-coating rates with a constant immersion time of 5 s and (**d**) various immersion times with a constant spin-coating rate of 2000 rpm.

**Figure 4:**
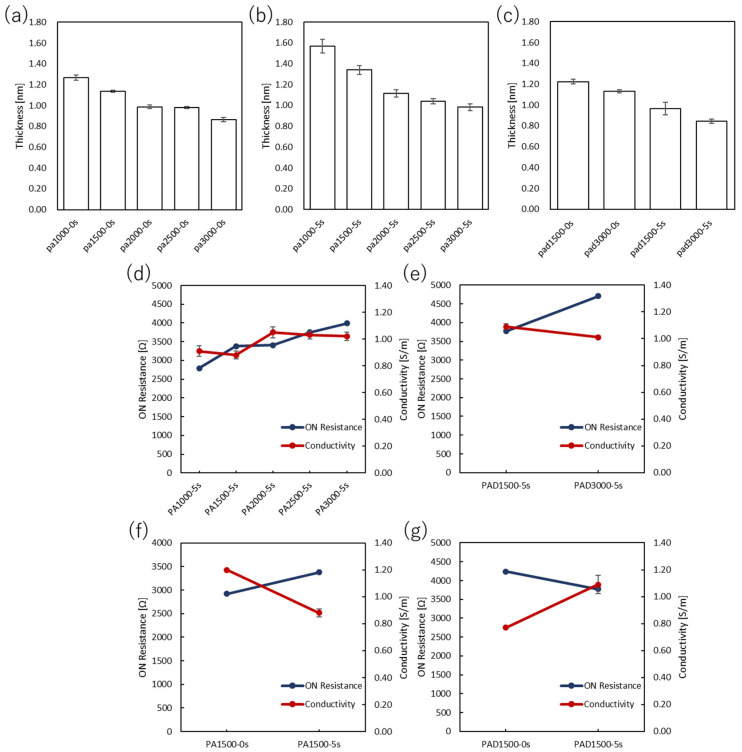
Film thicknesses of PANI OECTs at various spin-coating rates with the immersion time of (**a**) 0 s and (**b**) 5 s. (**c**) Film thicknesses of PANI:DBSA OECTs at various spin-coating rates with an immersion time of 5 s. The on-resistance and conductivity values of (**d**,**f**) PANI and (**e**,**g**) PANI:DBSA at (**d**,**e**) various spin-coating rates with a constant immersion time of 5 s and (**f**,**g**) various immersion times with a constant spin-coating rate of 1500 rpm.

The output curves of PEDOT:PSS devices and PANI devices are shown in Figure 1 and Figure 2, respectively. In the case of PANI devices, the V_D_ was set in the range of 0 V to 0.6 V, and the V_G_ was measured from 0 V to 0.6 V at intervals of 0.1 V. The values of on-resistance, resistivity, and conductivity were estimated from the OECT performances and are summarized in Figure 3 and Figure 4 and Tables S1 and S2.

The output characteristics of the PEDOT:PSS-based devices were optimized by changing the spin-coating rate. The results revealed that the resistance was higher than 1400 Ω when PEDOT:PSS films were prepared at a spin-coating rate of 1000 rpm and a rinse time in DI water of 5 s (P1000-5 s). This was also the case for the spin-coating rate of 3000 rpm (P3000-5 s). However, when the spin-coating rate was 2000 rpm (P2000-5 s), the lowest resistance value was obtained. On the other hand, the device (P3000-5 s) showed the highest conductivity of 18.1 S m^−1^. Conductivity increased with the increasing spin-coating rate, which is most likely associated with the film thickness and morphology. There is a previous study showing that PEDOT:PSS films crystallize when they are thermally annealed [20]. This implies that the thicker the film, the less uniform the crystallized PEDOT, and the thicker the films prepared at low spin-coating rates, the lower the conductivity. Overall, the film thickness is one of the significant parameters to optimize the electrical properties. Thinning the film thickness improves the calculated conductivity but may increase resistance due to the decrease in channel area. Therefore, a film thickness of about 150 nm is considered optimal.

The as-cast films were immersed in DI water to obtain the optimized surface morphology. The immersion time in DI water was thus one of the parameters needed to achieve high-performance OECTs. The device based on the as-cast film (P2000-0 s) gave the lowest resistance and highest conductivity. The conductivity decreased from P2000-0 s to P2000-15 min, but slightly increased when the immersion time was prolonged to 1 h (P2000-1 h). The conductivity of the device (P2000-18 h) was comparable to that of P2000-5 s. The purpose of immersing the films in DI water was to obtain a better film surface by partially dissolving oligomers and impurities. However, the conductivity of the device (P2000-5 s) was indeed lower than that of P2000-0 s. This result suggests that the as-cast film is sufficient for OECT applications.

The output characteristics of the PANI-based OECTs showed efficient conductivities, although the devices prepared at the larger spin-coating rates had higher on-resistance. The disadvantage of a thinner film is that the channel bandwidth to be stored is considerably narrower, resulting in lower conductivity. This effect also reduces durability against potential differences in loading between the source and drain electrodes. Specifically, the drain voltage could be applied up to about 1 V for PA1000-5 s. However, in the PA3000-5 s, the operation became unstable at around 0.6 V, and the drain current sharply dropped. For the film thickness, considering this issue, 3000 rpm is the upper limit of the spin-coating rate under the measurement conditions.

The immersion in DI water resulted in a decrease in conductivity even after about 5 s of immersion. As for the film thickness, the surface was somewhat scraped by immersion, resulting in a thin film. However, the conductivities of the devices prepared at high spin-coating rates were large, suggesting oxidation of the films. Therefore, this immersion operation in water was found to be unsuitable for PANI.

Similar to PANI, the conductivities of the PANI:DBSA devices with higher spin-coating rates were higher. When PAD1500-5 s with DBSA was compared with PA1500-5 s without DBSA, PAD1500-5 s had higher on-resistance and thinner films. In addition, the conductivity was slightly improved by increasing the spin-coating rate. When PAD3000-5 s and PA3000-5 s were compared, the conductivity was similar. Comparing PAD1500-0 s and PA1500-0 s, the conductivity of PAD1500-0 s was considerably lower. Since it was reported that the addition of DBSA to PANI decreases conducting and other related properties, the conductivity of PANI:DBSA without DI water washing was lower than that of the corresponding PANI film [32]. However, since the addition of DBSA most likely improved water resistance and chemical endurance, the conductivity increased when washed with DI water. This is because the negative impact of DI water immersion is considerably smaller than that of the PANI film.

### 3.2. Transfer Characteristics

**Figure 5:**
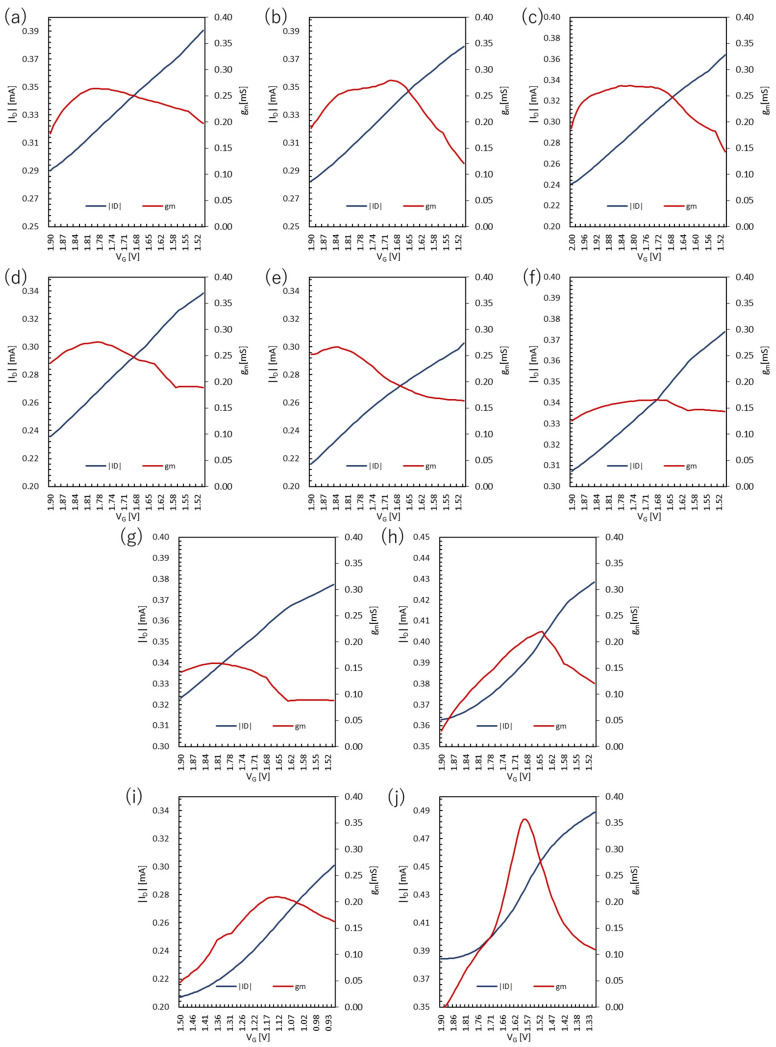
Transfer curves of the PEDOT:PSS devices: (**a**) P1000-5 s, (**b**) P1500-5 s, (**c**) P2000-5 s, (**d**) P2500-5 s, (**e**) P3000-5 s, (**f**) P2000-0 s, (**g**) P2000-5 min, (**h**) P2000-15 min, (**i**) P2000-1 h, and (**j**) P2000-18 h.

**Figure 6:**
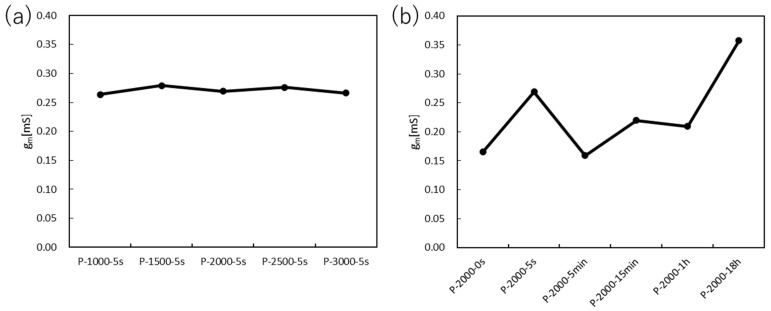
Transconductance of the PEDOT:PSS devices at (**a**) various spin-coating rates and (**b**) various immersion times in DI water.

The transfer curves and transconductance of the PEDOT:PSS devices are shown in Figure 5 and Figure 6, respectively. The highest transconductance of 0.280 mS was obtained for P1500-5 s when the spin-coating rate was optimized. The next highest value was 0.276 mS for P2500-5 s, and the other three types had the maximum transconductance in the range of 0.260~0.270 mS. The error from the average value of the maximum transconductance was within ±0.01 mS, indicating that the maximum transconductance hardly changes as the film thickness changes.

On the other hand, the immersion time in DI water showed a different behavior. The maximum transconductance increased with an immersion time short enough to momentarily wash the surface and decreased with a longer immersion time. The maximum value gradually increased with continued immersion for a longer period of time. Two sets of data, P2000-0 s and P2000-5 min, showed maximum values well below 0.20 mS. Therefore, it is likely that the transconductance is affected by the surface condition of the film due to the immersion in DI water.

**Figure 7:**
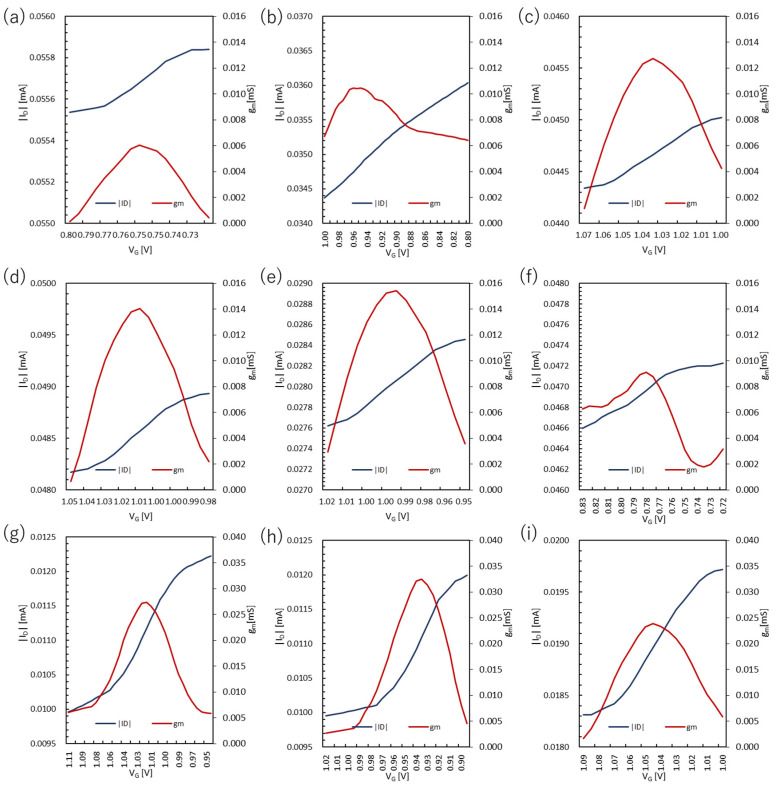
Transfer curves of the devices based on PANI and PANI:DBSA: (**a**) PA1000-5 s, (**b**) PA1500-5 s, (**c**) PA2000-5 s, (**d**) PA2500-5 s, (**e**) PA3000-5 s, (**f**) PA1500-0 s, (**g**) PAD1500-5 s, (**h**) PAD3000-5 s, and (**i**) PAD1500-0 s.

**Figure 8:**
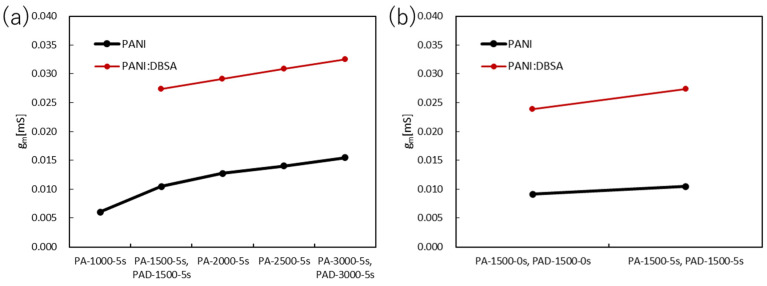
Transconductance of the PANI and PANI:DBSA devices at (**a**) various spin-coated rates and (**b**) various immersion times in DI water.

For PANI, the V_G_ was set from 1.5 V to 0 V, and the V_D_ was fixed at −0.2 V. The transfer curves and transconductance of the PANI devices are shown in Figure 7 and Figure 8, respectively. In the case of PANI, the maximum transconductance increased with increasing spin-coating rates, with PA3000-5 s having the highest value of 0.0154 mS. This may be because of the improved surface morphology of the film. However, as the spin-coating rate increased, the gate voltage durability of the PANI film became an issue. The highest applied voltage durability was 2.0 V for PA1000-5 s which tended to gradually decrease as the film became thinner. Samples of PANI films were prepared at spin-coating rates of 3500 and 4000 rpm, and measurements were attempted under the same conditions. However, the attempted measurement conditions did not provide data from which the transconductance could be calculated. This trend was also true for PANI:DBSA, with PAD3000-5 s being higher than PAD1500-5 s at 0.0325 mS. Furthermore, when compared with and without the addition of DBSA, the maximum transconductance more than doubled with the addition of DBSA. Similar to the PANI film, we prepared the PANI:DBSA film at 4000 rpm, but the transconductance could not be calculated.

When the PANI films were immersed in DI water, the transconductance values were higher than those of the films that were not immersed. The surface morphology of the film is thought to have a significant impact. PANI:DBSA films also improved the maximum transconductance by a factor of two or more.

### 3.3. Surface Morphology

**Figure 9:**
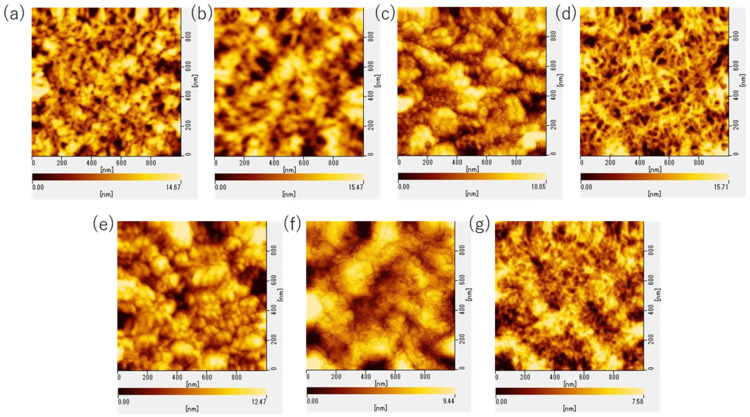
AFM images of PEDOT:PSS films: (**a**) P1000-5 s, (**b**) P2000-5 s, (**c**) P3000-5 s, (**d**) P2000-0 s, (**e**) P2000-5 min, (**f**) P2000-15 min, and (**g**) P2000-18 h.

**Figure 10:**
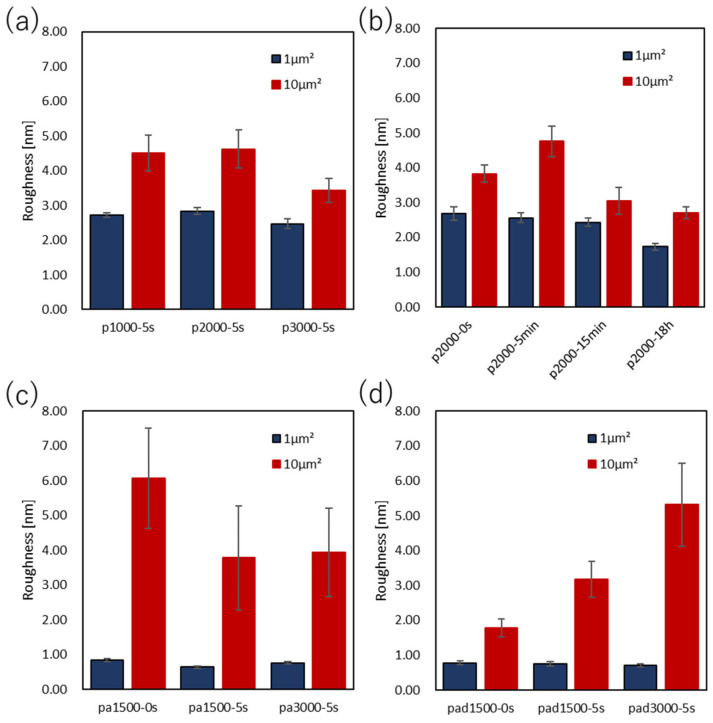
Surface roughness (Ra) of the PEDOT:PSS films at (**a**) various spin-coating rates and (**b**) various immersion times in DI water and the PANI and PANI:DBSA films at (**c**) various spin-coating rates and (**d**) various immersion times in DI water.

The surface morphology of the spin-coated films was observed by atomic force microscopy (AFM). Samples were prepared by spin-coating the polymer solutions on a glass substrate using the same procedure as for an electrode substrate, and the center area of the glass substrate was observed. The PEDOT:PSS samples observed were P1000-5 s, P2000-5 s, P3000-5 s, P2000-0 s, P2000-5 min, P2000-15 min, and P2000-18 h. The images in the range of 1μm^2^ are shown in Figure 9. The surface roughness of the samples is shown in Figure 10 and summarized in Table S3.

The surface morphology of P2000-5 s was rougher than that of P1000-5 s. In comparison, P3000-5 s had a slightly smoother surface. When the films were not immersed in DI water, the film surfaces were slightly smoother than those immersed shortly afterwards. Thus, immersion in DI water for short periods of time may slightly degrade the film surface. However, the longer the immersion time in DI water, the smoother the surface became. As a consequence, P2000-18 h had the smoothest surface.

**Figure 11:**
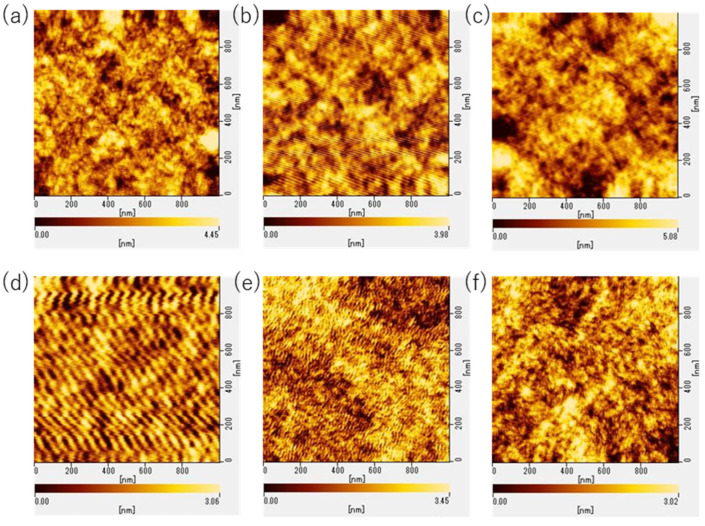
AFM images of the PANI and PANI:DBSA films: (**a**) PA1500-5 s, (**b**) PA3000-5 s, (**c**) PA1500-0 s, (**d**) PAD1500-5 s, (**e**) PAD3000-5 s, and (**f**) PAD1500-0 s.

The surface morphology of the PANI films was also observed using AFM. The samples observed were PA1500-5 s, PA3000-5 s, PA1500-0 s, PAD1500-5 s, PAD3000-5 s, and PAD1500-0 s. Images in the 1 μm^2^ range are shown in Figure 11. The surface roughness of the samples is shown in Figure 10 and summarized in Table S4.

Regarding the surface morphology of the PANI films, the surface roughness of both 1 μm^2^ and 10 μm^2^ became smoother by increasing the spin-coating rate. When immersed in DI water, the surface roughness decreased by nearly 0.2 nm, indicating that the surface became smoother. In PANI:DBSA, comparing PAD1500-5 s and PAD3000-5 s, the surface roughness at 10 μm^2^ was smoother on PAD1500-5 s. However, PAD-3000-5 s was smoother at 1 μm^2^. The sample without the DI water rinse had a slightly rougher surface at 1 μm^2^, but much smoother results in the 10 μm^2^ range. This may be due to the possibility that the samples with DBSA were more prone to mottling of the surface morphology due to washing.

## 4. Discussion

From the above results, the best OECT performance for PEDOT:PSS was obtained at a spin-coating rate of 2000 rpm and immersion in DI water for 18 h. For PANI, the best performance was obtained by adding DBSA, spin-coating at 3000 rpm, and washing the surface with DI water for a short time of about 5 s.

On-resistance and conductivity were higher for PEDOT:PSS, and conductivity was about 16 times higher than that of PANI, partly due to its thinner film. The maximum transconductance was 13 times higher for PEDOT:PSS. These results indicate that PEDOT:PSS considerably outperforms PANI in terms of performance as an OECT. This is thought to be because the presence of PSS considerably improved the oxidation resistance of the PEDOT:PSS film, allowing the formation of a relatively wide flow path even when the films were thin.

However, the uniformity of film thickness and the smoothness of the film surface are superior for PANI. In terms of film thickness, PEDOT:PSS showed a large difference in thickness depending on the location, whereas PANI showed a fairly uniform film thickness at 1μm^2^. Considering that PEDOT:PSS required various preparations to fabricate OECTs, PANI is superior in that better-morphology films can be easily formed in a shorter time. The surface of the PANI film was relatively easy to scrape, resulting in a large surface roughness value of 10μm^2^. In this regard, further surface smoothness and performance improvement can be expected by establishing a uniform cleaning method with DI water.

The main reason why PANI has not been more widely studied than PEDOT:PSS is because of its lower conductivity. However, when practical applications are considered, high reproducibility of film formation is extremely important. In the future, improving the conductivity of PANI will give it an advantage over PEDOT:PSS.

## 5. Conclusions

In this study, OECTs were fabricated using PEDOT:PSS and PANI, and their performances were evaluated. Regarding the optimization of fabrication conditions, in the case of PEDOT:PSS, a polymer film with a thickness of 150 nm was obtained by spin-coating at 3000 rpm and immersion in DI water for 18 h, based on the uniformity inside the film and the smoothness of the surface.

For PANI, the OECT performances and film surface morphology studies revealed that doping with DBSA improves the carrier concentration. Based on the effect of film surface smoothness, it was also found that the optimal conditions for PANI doped with DBSA were a spin-coating rate of 3000 rpm and surface washing with DI water for about 5 s, resulting in a polymer film with a thickness of 0.84 μm. The conductivity and maximum transconductance tended to increase with the increasing spin-coating rate, and further improvement in values may be expected by increasing the spin-coating rate higher than 3000 rpm. However, the higher oxidation rate, the shrinkage of channels formed by thinning, and the associated decrease in current and voltage tolerance had a greater impact, resulting in less stable OECT and less reliable data.

The basic performance of the PEDOT:PSS-based OECT is higher than that of the PANI-based OECT, and this is the reason why there are many PEDOT:PSS-based OECT studies. On the other hand, PANI is superior in terms of the smoothness of the polymer film, and research on biosensors using the PANI-based OECT is expected in the future.

## Figures and Tables

**Table 1:** Fabrication conditions of OECTs based on PEDOT:PSS.

Sample	Spin-Coating Rate (rpm) ^a^	Immersion Time ^b^
P1000-5 s	1000	5 s
P1500-5 s	1500	5 s
P2000-5 s	2000	5 s
P2500-5 s	2500	5 s
P3000-5 s	3000	5 s
P2000-0 s	2000	0 s
P2000-5 min	2000	5 min
P2000-15 min	2000	15 min
P2000-1 h	2000	1 h
P2000-18 h	2000	18 h

^a^ Spin-coating speed of the PEDOT:PSS solution. ^b^ Time to soak the spin-coated films in DI water.

**Table 2:** Fabrication conditions of OECTs based on PANI.

Sample	Spin-Coating Rate (rpm) ^a^	Immersion Time ^b^
PA1000-5 s	1000	5 s
PA1500-5 s	1500	5 s
PA2000-5 s	2000	5 s
PA2500-5 s	2500	5 s
PA3000-5 s	3000	5 s
PA1500-0 s	1500	0 s
PAD1500-5 s	1500	5 s
PAD3000-5 s	3000	5 s
PAD1500-0 s	1500	0 s

^a^ Spin-coating speed of the PANI solution. ^b^ Time to soak the spin-coated films in DI water.

## Data Availability

The data presented in this study are available on request from the corresponding author.

## Supplementary Materials

The following supporting information can be downloaded at: https://www.mdpi.com/article/10.3390/polym15244657/s1, Table S1: OECT characteristics based on PEDOT:PSS; Table S2: OECT characteristics based on PANI; Table S3: Surface roughness data of PEDOT:PSS films; Table S4: Surface roughness data of PANI films.
