# Supplementary material for: PEDOT:PSS versus Polyaniline: A Comparative Study of Conducting Polymers for Organic Electrochemical Transistors
Source: Polymers (Basel). 2023 Dec 10;15(24):4657. doi: 10.3390/polym15244657 (PMC10747145; doi:10.3390/polym15244657)
Supplement: Supplementary file 1 [file polymers-15-04657-s001.zip › polymers-2753118-supplementary.pdf]

Supporting Information

for

PEDOT:PSS versus Polyaniline: A Comparative Study of Conducting Polymers for Organic Electrochemical Transistors

*Ryotaro Kawamura and Tsuyoshi Michinobu**

Department of Materials Science and Engineering, Tokyo Institute of Technology, 2-12-1 Ookayama, Meguro-ku, Tokyo 152-8552, Japan.

*E-mail: michinobu.t.aa@m.titech.ac.jp

General Measurements

A patterned electrode (BAS 0011598) was used as the substrate electrode of OECTs. A ultraviolet ozone cleaner (TECHNOVISION, INC. UV-208) was used to clean the substrate of OECTs. A spin coater used was MIKASA MS-B100. For thermal annealing, a digital hotplate of CORNING PC-400D was used. Output and transfer characteristics were measured using a parameter analyzer, KEITHLEY 4200A-SCS. Film thickness measurements were done with a surface prolifometer, KLA-Tencor D-100. Atomic force microscopy (AFM) was measured by using an SPI3800N and SPA300 with a stiff cantilever DF-20.

Supporting Tables

Table S1 Characteristics of OECTs based on PEDOT:PSS

Sample	P1000-5s	P1500-5s	P2000-5s	P2500-5s	P3000-5s	P2000-0s	P2000-5min	P2000-15min	P2000-1h	P2000-18h
**ON Resistance [Ω]**	1400	1200	1160	1300	1470	940	1310	1350	1310	1080
**Resistivity [Ω・m]**	0.12	0.090	0.067	0.065	0.055	0.052	0.075	0.078	0.075	0.062
**Conductivity [S/m]**	8.16 ±0.23	11.1 ±0.4	15.0 ±1.6	15.4 ±1.4	18.1 ±1.7	19.3 ±1.7	13.3 ±1.4	12.9 ±1.4	13.3 ±1.4	16.1 ±1.8
**Thickness [nm]**	350 ±10	300 ±11	230 ±25	200 ±18	150 ±14	220 ±19	230 ±25	230 ±25	230 ±25	230 ±25

Table S2 Characteristics of OECTs based on PANI

Sample	PA1000-5s	PA1500-5s	PA2000-5s	PA2500-5s	PA3000-5s	PA1500-0s	PAD1500-5s	PAD3000-5s	PAD1500-0s
**ON Resistance [Ω]**	2790	3380	3410	3750	3990	2920	3770	4710	4240
**Resistivity [Ω・m]**	1.10 ±0.04	1.13 ±0.03	0.96 ±0.03	0.98 ±0.03	0.98 ±0.03	0.83 ±0.01	0.91 ±0.06	0.99 ±0.02	1.29 ±0.02
**Conductivity [S/m]**	0.91 ±0.04	0.88 ±0.03	1.05 ±0.04	1.03 ±0.03	1.02 ±0.03	1.20 ±0.01	1.09 ±0.07	1.01 ±0.02	0.77 ±0.01
**Thickness [μm]**	1.57 ±0.06	1.34 ±0.04	1.12 ±0.04	1.04 ±0.03	0.98 ±0.03	1.14 ±0.01	0.97 ±0.06	0.84 ±0.02	1.22 ±0.02

Table S3 Surface roughness (Ra) of PEDOT:PSS films

Sample	P1000-5s	P2000-5s	P3000-5s	P2000-0s	P2000-5min	P2000-15min	P2000-18h
1μm^2[nm]	2.72 ±0.07	2.84 ±0.09	2.47 ±0.14	2.68 ±0.20	2.56 ±0.14	2.43 ±0.12	1.73 ±0.10
10μm^2[nm]	4.51 ±0.52	4.62 ±0.54	3.44 ±0.34	3.83 ±0.24	4.76 ±0.43	3.05 ±0.39	2.71 ±0.16

Table S4 Surface roughness (Ra) of PANI films

Sample	PA1500-5s	PA3000-5s	PA1500-0s	PAD1500-5s	PAD3000-5s	PAD1500-0s
1μm^2[nm]	0.652 ±0.03	0.764 ±0.03	0.841 ±0.05	0.761 ±0.07	0.708 ±0.04	0.784 ±0.06
10μm^2[nm]	3.78 ±1.49	3.94 ±1.28	6.07 ±1.45	3.17 ±0.52	5.31 ±1.19	1.78 ±0.25

Supporting Figure

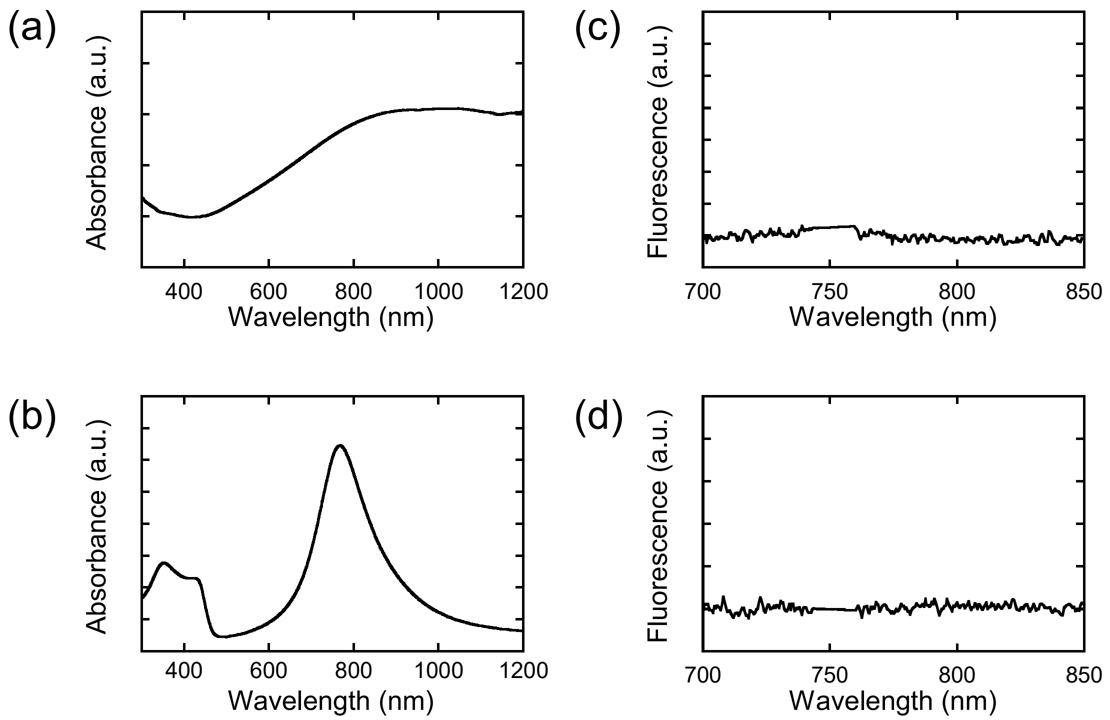

Figure S1 (a,b) Absorption and (c,d) fluorescence (excited at 750 nm) spectra of the thin films of (a,c) PEDOT:PSS and (b,d) PANI.
